# The Seroprevalence of Hepatitis C Virus (HCV) in Hemodialysis Patients in Oman: A National Cross-Sectional Study

**DOI:** 10.1007/s44197-023-00149-6

**Published:** 2023-09-12

**Authors:** Intisar Al Shukri, Adil Al Wahaibi, Hanan Al kindi, Yaqoub Al-Maimani, Amal Al Maani, Abdullah Alqayoudhi, Mersum C. Methew, Jini Pradeesh, Raiya Al Abrawi, Abdo Debs, Nabila Mansoor, Ahmed AlRahbi, Wadha Al Balushi, Mahmood Alharrasi, Badriya Al Mamari, Magda Fakhry Soliman, Afraa Alsenaidi, Mohammed Al Alawi, Omaima Al Ismaili, Seif Al-Abri, Amina Al-Jardani

**Affiliations:** 1 Directorate General for Disease Surveillance and Control, Central Public Health Laboratories, Ministry of Health, Muscat, Oman; 2 Department of Surveillance, Directorate General for Disease Surveillance and Control, Ministry of Health, Muscat, Oman; 3 Bawshar Dialysis Center, Ministry of Health, Muscat, Oman; 4 Directorate General for Disease Surveillance and Control, Ministry of Health, Muscat, Oman; 5 Department of Infection Prevention and Control, Directorate General for Disease Surveillance and Control, Ministry of Health, Muscat, Oman; 6 Royal Hospital, Ministry of Health, Muscat, Oman; 7 Quriyat Hospital, Ministry of Health, Muscat, Oman; 8 Salalah Dialysis Unit, Ministry of Health, Muscat, Oman; 9 Ibra Dialysis Unit, Ministry of Health, Muscat, Oman; 10 Sohar Hospital, Ministry of Health, Sohar, Oman; 11 As Seeb Dialysis Center, Ministry of Health, Muscat, Oman; 12 Shinas Renal Dialysis Unit, Ministry of Health, Muscat, Oman; 13 Al Buraymi Dialysis Unit, Ministry of Health, Muscat, Oman; 14 Jalan Dialysis Unit, Ministry of Health, Muscat, Oman; 15 Al Rustaq Dialysis Unit, Ministry of Health, Muscat, Oman

**Keywords:** Hepatitis C virus, Seroprevalence, Dialysis unit, Oman, Cross-sectional

## Abstract

**Background:**

HCV infection in hemodialysis units is a significant cause of morbidity and mortality. The risk of HCV infection among dialysis patients is higher compared to the general population due to high potential blood exposures in hemodialysis settings. This study aims to assess the national HCV seroprevalence in selected dialysis units and to determine the risk factors for acquiring HCV infection.

**Methods:**

This cross-sectional study was conducted from 1 January to 31 March 2021. A total of 734 patients from 11 hemodialysis centers in Oman were included. Samples were tested simultaneously for HCV antibodies and HCV RNA. HCV genotyping was determined in all viremic patients. Demographic and hemodialysis center related data were gathered and their association with the positive HCV serology were explored using univariate and multivariate logistic regression analysis.

**Results:**

Out of 800 patients selected from 11 dialysis units for the study, 734 patients (91.8%) were included. The overall seroprevalence of HCV infection among hemodialysis patients was 5.6%. (41/734). HCV RNA was detected in 31.7% (13/41) of seropositive hemodialysis patients. The most common genotype was subtype 1a, followed by subtype 3. Variables associated with high HCV prevalence were family history of HCV and duration of dialysis.

**Conclusion:**

The prevalence of infection within hemodialysis patients in Oman has significantly decreased but remained higher than the general population. Continuous monitoring and follow-up, including periodic serosurvey and linkage to care and treatment are recommended. Additionally, practice audits are recommended for identifying gaps and ensuring sustainability of best practices and further improvement.

**Supplementary Information:**

The online version contains supplementary material available at 10.1007/s44197-023-00149-6.

## Introduction

HCV infection is a major cause of chronic hepatitis and is a significant public health problem [1, 2]. In 2019, globally, around 58 million people have chronic HCV infection, with almost 1.5 million new infections occurring per year [3]. In 2019, World Health Organization estimated that, approximately 290,000 people died from hepatitis C, mostly cirrhosis and hepatocellular carcinoma [2, 3].

In May 2016, the World Health Assembly adopted the first Global health sector strategy on viral hepatitis, 2016–2020. The strategy proposed the elimination of viral hepatitis as a public health threat by 2030. Here elimination is defined as a 90% reduction in new chronic infections and a 65% reduction in mortality, compared to the 2015 baseline. In May 2022, the 75th World Health Assembly noted a new set of integrated global health sector strategies on HIV, viral hepatitis and sexually transmitted infections for 2022–2030 [3].

HCV infection is associated with increased extra-hepatic complications including increased incidence and rapid progression of chronic Kidney diseases, as well as higher mortality in chronic kidney disease and renal transplant patients [4, 5].

Patients on hemodialysis are at high risk of acquiring HCV infection if strict infection control measures are not implemented in the dialysis unit. The risk factors for acquiring HCV infection in dialysis units include risks associated with dialysis unit like the number of patients in relation to the number of staff, duration of dialysis, use of multi-dose medication vials, number of blood transfusions, history of organ transplant, previous surgery and history of intravenous drug use [5, 6]. HCV treatment is now available and antiviral medicines can cure 90–100% of persons with hepatitis C infection [7].

As per national and international guidelines, hemodialysis patients undergo surveillance for testing blood-borne viruses, including HCV infection. The Renal Association recommends screening all new patients starting hemodialysis, followed by HCV antibody testing every 3 months [8, 9]. It is recommended to screen using HCV nucleic acid amplification testing in hemodialysis patients with identified risk factors [8, 9]. In Oman, HCV antibody testing is done annually or more frequently if risk factors are found, or the liver function test is deranged. As of the 2019 statistical report, 2076 patients undertook hemodialysis in 23 dialysis centers throughout Oman [10].

Based on the Dialysis Outcomes and Practice Patterns Study (DOPPS), the overall prevalence of HCV is 9.9% among adult hemodialysis patients randomly selected from dialysis facilities in high- and middle-income countries [11]. In the Middle East, the overall prevalence was 25.3% [12]. In 1993, a seroprevalence study was conducted in a tertiary care hospital in Oman, and it showed that the HCV prevalence in patients on hemodialysis was 26.5% [13]. No recent national study reported the prevalence of HCV infection in Omani patients undergoing hemodialysis.

This study aims to assess the prevalence of HCV infection among hemodialysis patients in different institutions and its determinants in Oman.

## Methods

This is a cross-sectional observational study involving randomly selected hemodialysis patients from different hemodialysis units in Oman during the period from 1st of January to 31st of March 2021.

The patients were selected using stratified random sampling by governorates. Patients in each institution were selected randomly using computer-based randomization from the sample frame. The sample size was calculated according to the governorate population of hemodialysis patients aiming for a total of 800 for the entire study. It was calculated based on 95% confidence interval (CI) and 5% margin of error samples and estimated prevalence of HCV in hemodialysis patients as 26% [13].

Blood samples collected at dialysis centers were tested simultaneously for HCV antibodies and RNA at the Central Public Health Laboratories. The HCV antibody was tested using electrochemiluminescence anti-HCV test (Elecsys Anti-HCV II Roche Diagnostics, Tokyo, Japan) according to the manufacture recommendations. HCV RNA was tested using AmpliPrep/cobas TaqMan HCV Qualitative test (Roche Molecular Diagnostic, Meylan, France). HCV genotyping was performed in all viremic samples using Roche cobas® HCV GT (Roche Molecular Diagnostics, Pleasanton, CA).

All patients who were included in the study were asked to fill out an online questionnaire with a staff nurse looking after the patient during the dialysis session. The following information was collected: age, sex, nationality, hospital, hemodialysis unit ame, region, date of diagnosis, duration of dialysis, previous surgery, blood transfusion history of transplant, history of intravenous drug use and other risk factors. A dialysis unit-related form was filled out by the in-charge nurse of the dialysis unit. In this form, risk factors related to the unit were included, such as the number of staff, the number of isolation units, and number of patients per session. Only patients with a completed questionnaire and those who consented were included. Patients who could not attend the interview because of death, transplant or prolonged hospital admission were excluded.

### Statistical Analysis

Sample characteristics and demographic features were presented as sums and percentages from the total. Age was categorized into 0–29, 30–59, ≥ 60-year-old. Positive cases were assigned if the HCV IgG levels were ≥ 1. Samples with a cut-off ≤ 0.9 are considered negative. Samples with cut-off index > 0.9 and < 1 are released as equivocal results were considered negative after verification of the PCR for negative HCV RNA results and repeat negative serology. Seroprevalence classified by gender, age groups, institutions, and governorate was presented with 95% CI calculated using a design-based likelihood method in the survey package in R software (R Project, Vienna, Austria).^1^ Crude odds ratio (COR) with 95% CI of the relation between seropositivity and different variables were calculated using univariate binary logistic regression analysis. Multivariable logistic regression was also performed to calculate the adjusted odds ratios. Variables for the multivariable analysis were chosen using stepwise regression based on the Akaike information criterion method.

All statistical analysis was done with R software version 4.0 (R Project, Vienna, Austria).^2^

## Results

Out of 800 patients selected from 11 dialysis units for the study, 779 (97.4%) signed informed consent, and 45 patients were excluded either because the online questionnaires were not filled out properly (*N* = 41) or because of leaked samples (*N* = 4), Supplementary Fig. 1. A total of 734 patients (91.8%) were included in the study. In the study population, most of the dialysis patients were 30 years old and older (Table 1).

**Table 1:** Sociodemographic characteristics of the studied sample

	Overall (*N* = 734)
Age group	
0–29-year-old	64 (8.7%)
30–59-year-old	355 (48.5%)
60 years and above	313 (42.8%)
Male	425 (58.1%)
Institute	
Al Buraymi Hospital	34 (4.6%)
Al Rustaq Hospital	70 (9.5%)
As Seeb Dialysis Center	115 (15.7%)
As Sultan Qaboos Hospital	13 (1.8%)
Bawshar Dialysis Center	244 (33.2%)
Ibra Hospital	39 (5.3%)
Jalan dialysis unit	78 (10.6%)
Khasab Hospital	15 (2.0%)
Qurayyat Hospital	21 (2.9%)
Shinas dialysis unit	26 (3.5%)
Sohar dialysis unit	79 (10.8%)
HCW	16 (2.2%)
Knowledge about hepatitis	383 (52.8%)
Family history	48 (6.6%)
History of blood transfusion	361 (49.9%)
Previous surgery	597 (82.9%)
Dialysis duration (years)	
Mean (SD)	5.0 (4.7)
Range	0.0–32.0
Frequency of dialysis	
Number missing	9
Three times a week and more	625 (86.2%)
Less than three times a week	100 (13.8%)
History of drug injection or cupping	156 (21.5%)
History of needle stick injury	5 (0.7%)
History of organ transplant	37 (5.1%)
Extramarital relationships	11 (1.6%)
History of imprisonment	19 (2.6%)
Seropositive	41 (5.6%)
Active infection (HCV RNA)	14 (1.9%)

Most of the patients were Omani 722 (98.6%), with close to equal distribution between the two genders (58.1%) males and (41%) females. The highest number of patients who were selected in this study were from the Bawshar Dialysis Center, followed by the As Seeb Dialysis Center, 33.2 and 15.7, respectively.

Regarding knowledge about hepatitis, 52.8% of the study group had previous knowledge about hepatitis. Six percent of the patients involved in the study gave a family history of HCV infection. History of blood transfusion either before or after initiation of the hemodialysis was reported by 49.9% of the patients. History of surgical intervention was reported by 82.9% of patients.

The overall seroprevalence of HCV infection among hemodialysis patients was 5.6% (see Table 1).

**Fig. 1:**
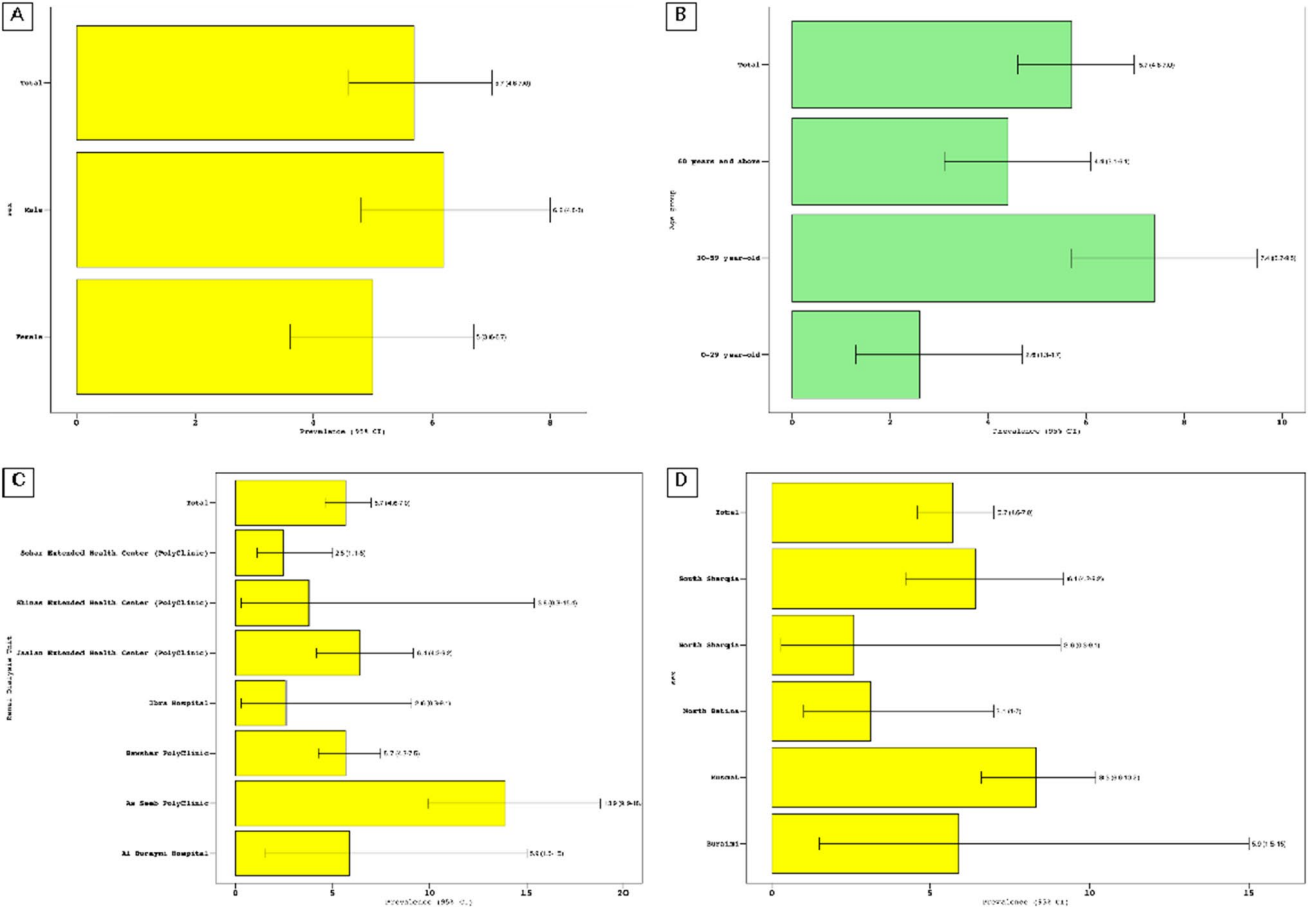
Seroprevalence of HCV in hemodialysis classified by **A** Sex, **B** Age groups, **C** Dialysis units, **D** Governorates

The difference in seropositivity between males and females was not statistically significant at 6.2% (95% CI = 4.8–8.0%), 5.0% (95% CI = 3.6–6.7%), respectively. On the other hand, there was a statistically significant difference between the age group 30–59 and 0–29-year-old, 7.4 (5.7–9.5), 2.6 (1.3–4.7), respectively (Fig. 1A and B).

The institute where the patient was dialyzed also had an impact on the HCV antibody positive result as it was noted that As Seeb Dialysis Center had the highest seroprevalence compared to the rest of the dialysis units, 13.9% (9.9–18.0%). Nevertheless, the Bawshar Dialysis Center which belongs to the same region, Muscat, is significantly lower than As Seeb in seroprevalence, it has a positivity of 5.7% (95% CI = 4.3–7.5%) (Fig. 1C).

The univariate analysis shows that seropositivity is positively associated with knowledge about hepatitis, crude odds ratio (COR) (95% CI); 2.56 (1.3–5.41). Patients with a family history of HCV infection have a 20 times greater chance to be seropositive than those with no family history, COR (95% CI); 20.44 (9.91–42.42). In addition, patients with longer duration of dialysis have more risks of being seropositive for HCV, COR (95% CI); 1.09 (1.03–1.15). Compared to other dialysis centers, As Seeb Dialysis Center has four times the risk of being seropositive, COR (95% CI); 3.84 (1.95–7.39) (Table 2). The multivariable analysis using stepwise regression analysis selected family history, dialysis duration and being from As Seeb Dialysis Center and gave almost similar associations (Table 3).

**Table 2:** Univariate analysis^1^ of the relation between seropositivity and different variables

Variable	*P*-value	COR (95% CI)
Age group 0–29-year-old: 60 years and above	0.67	0.69 (0.11–2.55)
Age group 30–59-year-old: 60 years and above	0.16	1.62 (0.84–3.25)
Omani: non-Omani	0.98	942,299.25 (0–inf.)
Sex male: Female	0.47	1.27 (0.67–2.49)
Knowledge about hepatitis	< 0.05	2.56 (1.3–5.41)
Family history	< 0.05	20.44 (9.91–42.42)
History of blood transfusion	0.43	0.78 (0.41–1.46)
surgery	0.20	1.97 (0.77–6.66)
Dialysis duration (in years)	< 0.05	1.09 (1.03–1.15)
Frequency of dialysis three times a week and more: < three times a week	0.11	3.17 (0.95–19.69)
History of drug injection or cupping	0.27	0.61 (0.23–1.37)
History of organ transplant	0.51	1.5 (0.35–4.43)
As Seeb Dialysis Center*	< 0.05	3.84 (1.95–7.39)
Al Buraymi Hospital*	0.93	1.06 (0.17–3.67)
Al Rustaq Hospital*	0.98	–
As Sultan Qaboos Hospital*	0.98	–
Ibra Hospital*	0.41	0.43 (0.02–2.07)
Jalan Dialysis Unit*	0.73	1.18 (0.4–2.85)
Khasab Hospital*	0.98	–
Qurayyat Hospital*	0.98	–
Shinas Dialysis Unit*	0.69	0.67 (0.04–3.28)
Bawshar Dialysis Center*	0.89	1.04 (0.52–2)
Sohar Dialysis Unit*	0.22	0.41 (0.07–1.37)

^1^COR was calculated using binary logistic regression, the outcome variable is seropositivity and the predictors are the different variables

*Analysis done using dummy variable method

**Table 3:** Multivariable analysis^1^ of the relation between seropositivity and different variables

variable	*P*-value	Adjusted odds ratio (95% CI)
Family history	< 0.05	22.90 (10.35–52.87)
Dialysis duration	0.087	1.05 (1.0–1.12)
As Seeb Dialysis Center*	< 0.05	5.16 (2.3–11.77)

^1^Adjusted odds ratio was calculated using binary logistic regression, the outcome variable is seropositivity, and the predictors were selected from the different variables using forward and backward stepwise regression based on Akaike information criterion

*Analysis was done using the dummy variable method

**Fig. 2:**
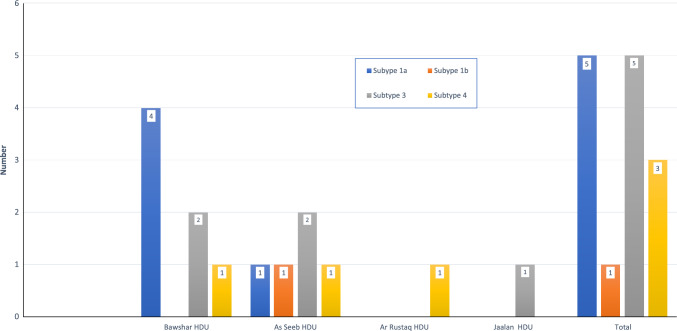
Distribution of hepatitis C subtypes among patients in the included renal dialysis units

Active infection (HCV RNA detected) was found in 31.7% (13/41) of the seropositive hemodialysis patients. One patient tested negative for HCV antibodies, but HCV RNA was detected. Three patients were diagnosed to have HCV infection for the first time during this study, two of them tested positive by serology and PCR and one tested positive only by PCR. All viremic patients were genotyped. Subtype 1a was the most prevalent genotype, followed by subtype 3. Three patients had genotype 4 out of 14 viremic patients (Fig. 2).

## Discussion

Our study is the first national multi-center study to assess the prevalence of HCV among dialysis patients. The overall seroprevalence of HCV in dialysis units is 5.7% which is much lower than the result of the previous study (26.5%) conducted in 1993 [13]. This seroprevalence was comparable to the seroprevalence of neighboring countries such as Saudi Arabia and Kuwait [14, 15], but much less than countries in Pakistan (32.33%), Sudan (17%) and other countries in the Middle East like Egypt (50%) and Syria (25.3%) [12, 16, 17]. This reduction may reflect the fact that in 1990, HCV screening of blood donation was introduced in Oman, and infection control practices and adherence to infection control guidelines were improved. Additionally, Oman used to import blood from the USA from mid-1970 to the early 1990s, during which HCV was the most common cause of chronic blood-borne infection in the USA [18]. While in 1990, Oman relied on local blood donation to meet its demand. Currently all blood donors are subjected to screening for blood borne viruses including HCV with serology and NAAT [24].

Despite all these measures, HCV prevalence in the hemodialysis unit is still higher than in the general population, which is less than 1% for blood donors [13]. This is because these patients are at high risk of infection as a result of a number of factors, including the frequent and prolonged potential exposure to blood, the close distance of dialysis patients to each other, health care staff moving between patients, frequent hospitalization and surgery, and more importantly, non-adherence of health care workers to the recommended infection control practices [5, 6, 11].

In the current study, blood transfusion was not identified as a significant factor associated with HCV positivity in hemodialysis units suggesting that health care-associated transmission was more to blame. Hemodialysis units have a unique feature of a high possibility of blood contamination of surfaces, environment, drugs, devices, and machines, and also, a large number of patients are treated at the same time and place [5]. In our study, As Seeb Dialysis Center had the highest seroprevalence compared to other units. As Seeb Dialysis Center, when compared to Bawshar Dialysis Center (both in the same district), share many features like a large number of patients, high patients to staff ratio and it received patients for temporary dialysis from different centers all over the country. However, the As Seeb Dialysis Center has higher seroprevalence than the Bawshar Dialysis Center (13.9% and 5.7%, respectively), which can be explained by the fact that it is a dedicated unit for the known infected cases as they have more isolation rooms. Nevertheless, more attention is needed for evaluation of infection prevention and control practices, including the failure to adhere to good hand hygiene and the proper use of gloves which both were described in different studies as a cause for outbreaks in dialysis units [6].

Healthcare-associated transmission of hepatitis C in different healthcare settings including hemodialysis was reported in different countries [4]. The duration of dialysis is reported in different studies as a major risk factor for acquiring HCV infection, and this is similar to the current study [5, 11]. Knowledge about hepatitis was associated with HCV positivity since the majority of the seropositive patients were aware that they had the infection and only three new patients were identified during the study. Newly detected cases of HCV infection are not uncommon in hemodialysis units, as reported by DOPPS. This study depicted seroconversion rates between 1.1 and 3.6% per 100 patient years in participating countries in 2004 and a rate between 1.2 and 2.9% per 100 patient years in DOPPS 5 (2012–2015) [11, 19]. Interestingly, a family history of HCV infection was identified as a significant factor of HCV positivity, although the vertical and sexual HCV transmission is not considered a common mode of transmission.

Of HCV seropositive patients, 31.7% were viremic, which is considered to be high, in comparison to studies from Sudan and Brazil [17, 20] with the advent of direct-acting antiviral treatment and the high success rate of cure. The patient link to HCV care should be improved and streamlined. HCV genotype 1a and 3 are both the most common genotypes detected, followed by genotype 4 similar to the findings from previous studies in Oman and other countries [14, 18, 20, 21].

Identifying the prevalence of HCV infection among hemodialysis patients will help to plan a strategy for preventing transmission in the setting of dialysis including infection control measures [22]. It may also lead to changes in the current practice of the surveillance, including more frequent testing and the use of HCV nucleic acid amplification testing based on data from prevalence studies in areas with high HCV prevalence [8].

HCV infection is a treatable disease with a high success rate with the advent of direct-acting antivirals; hemodialysis patients identified to have active HCV infection will be referred to a specialist for treatment and enhanced infection control measures will be followed during dialysis sessions to prevent further transmission of HCV infection. The high number of HCV viremic patients indicate the need to establish an integrated care pathway with muti disciplinary team involvement to improve the management of these patients and to prevent further transmission of HCV infection [23].

## Conclusion

The prevalence of hepatitis C infection among hemodialysis patients in Oman have significantly decreased compared to previous studies from the 1990s owing to an overall improvement in infection prevention and control practices, screening programs and safety of blood products. Continuous monitoring and follow-up, including periodic serosurveys and linkage to care and treatment is recommended. In addition, practice audits are recommended for identifying gaps and ensuring the sustainability of best practices and further improvement.

### Supplementary Information

Below is the link to the electronic supplementary material.

Supplementary file1 (DOCX 47 KB)

## Data Availability

The data presented in this study are available on request from the corresponding author upon reasonable request.

## Abbreviations

CI: Confidence interval
COR: Crude odds ratio
DOPPS: Dialysis Outcomes and Practice Patterns Study
HCV: Hepatitis C virus
NAAT: Nucleic Acid Amplification Test
PCR: Polymerase chain reaction
RNA: Ribonucleic acid

## Footnotes

1 T. Lumley (2020) "survey: analysis of complex survey samples." R package version 4.0.

2 R Core Team (2020). R: A language and environment for statistical computing. R Foundation for Statistical Computing, Vienna, Austria. URL https://www.R-project.org/.
