# Supplementary material for: The Seroprevalence of Hepatitis C Virus (HCV) in Hemodialysis Patients in Oman: A National Cross-Sectional Study
Source: J Epidemiol Glob Health. 2023 Sep 12;13(4):774–81. doi: 10.1007/s44197-023-00149-6 (PMC10686920; doi:10.1007/s44197-023-00149-6)
Supplement: Supplementary file 1 — Supplementary file1 (DOCX 47 KB) [file 44197_2023_149_MOESM1_ESM.docx]

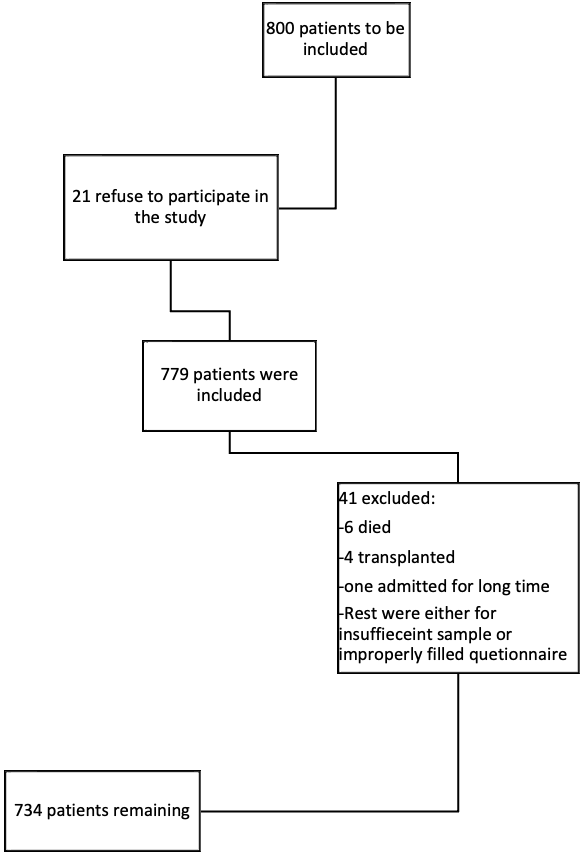


**Supplementary Fig. 1.** Participant flowchart of the number of samples selected, included, and excluded from the study
